# The effect of dosage on the protective efficacy of whole-sporozoite formulations for immunization against malaria

**DOI:** 10.1038/s41541-023-00778-9

**Published:** 2023-11-24

**Authors:** Diana Moita, Catarina Rôla, Helena Nunes-Cabaço, Gonçalo Nogueira, Teresa G. Maia, Ahmad Syibli Othman, Blandine Franke-Fayard, Chris J. Janse, António M. Mendes, Miguel Prudêncio

**Affiliations:** 1 Instituto de Medicina Molecular João Lobo Antunes, Faculdade de Medicina da Universidade de Lisboa, Lisboa, Portugal; 2 Faculty of Health Sciences, Universiti Sultan Zainal Abidin, 21300 Terengganu, Malaysia; 3 Department of Parasitology, Leiden University Medical Center, Leiden, Netherlands

**Keywords:** Live attenuated vaccines, Malaria

## Abstract

Immunization with *Plasmodium* sporozoites, either attenuated or administered under the cover of an antimalarial drug, can induce strong protection against malaria in pre-clinical murine models, as well as in human trials. Previous studies have suggested that whole-sporozoite (WSpz) formulations based on parasites with longer liver stage development induce higher protection, but a comparative analysis of four different WSpz formulations has not been reported. We employed a rodent model of malaria to analyze the effect of immunization dosage on the protective efficacy of WSpz formulations consisting of (i) early liver arresting genetically attenuated parasites (EA-GAP) or (ii) radiation-attenuated sporozoites (RAS), (iii) late arresting GAP (LA-GAP), and (iv) sporozoites administered under chemoprophylaxis, that are eliminated upon release into the bloodstream (CPS). Our results show that, unlike all other WSpz formulations, EA-GAP fails to confer complete protection against an infectious challenge at any immunization dosage employed, suggesting that a minimum threshold of liver development is required to elicit fully effective immune responses. Moreover, while immunization with RAS, LA-GAP and CPS WSpz yields comparable, dosage-dependent protection, protection by EA-GAP WSpz peaks at an intermediate dosage and markedly decreases thereafter. In-depth immunological analyses suggest that effector CD8+ T cells elicited by EA-GAP WSpz immunization have limited developmental plasticity, with a potential negative impact on the functional versatility of memory cells and, thus, on protective immunity. Our findings point towards dismissing EA-GAP from prioritization for WSpz malaria vaccination and enhance our understanding of the complexity of the protection elicited by these WSpz vaccine candidates, guiding their future optimization.

## Introduction

Malaria, caused by protozoan parasites of the genus *Plasmodium*, is the most prevalent parasitic infection worldwide, accounting for ~600,000 of a total of ~725,000 yearly deaths caused by mosquito-borne diseases^1^. The World Health Organization (WHO) estimated that in 2021 there were 247 million cases of malaria globally, representing a 2.5% increase relative to 2020, most of which occurring in countries in the WHO African region^1^. This resurgence in the incidence of malaria cases, following a period of steady decrease between 2000 and 2019^2^, highlights the urgent need for investment in malaria control and elimination measures, with a highly efficacious vaccine potentially constituting the ultimate step on the road towards disease elimination.

Mammalian infection by *Plasmodium* parasites begins when an infected *Anopheles* mosquito injects hepatotropic sporozoites (spz) into the host’s skin during a blood meal. Spz then travel to the liver and productively invade hepatocytes in a phase of asexual replication that culminates in the release of thousands of red blood cell (RBC)-infectious merozoites into the bloodstream^3,4^. There, parasites rapidly infect RBCs and undergo successive cycles of invasion, replication and erythrocyte burst. This blood stage of *Plasmodium* infection is responsible for the emergence of clinical symptoms^5^ and for parasite transmission upon ingestion of intraerythrocytic gametocytes by the mosquito vector^6^.

The most efficacious malaria vaccines reported to date are designed to target the pre-erythrocytic (PE) stages, i.e. spz and liver stages, of the *Plasmodium* life cycle (reviewed in ref. ^7^). Among these, the subunit vaccine RTS,S/AS01 (RTS,S), which specifically targets the circumsporozoite protein of *P. falciparum* (*Pf*), is the first malaria vaccine to be endorsed by the WHO for administration to children living in regions of moderate-to-high malaria transmission^8^. Nonetheless, the efficacy of RTS,S remains relatively limited and of short duration^9^, which does not fulfill the WHO’s goal of having a licensed vaccine affording at least 75% protection against malaria by 2030^10^. An alternative to vaccination based on the delivery of specific *Plasmodium* antigens is the use of whole-sporozoite (WSpz) immunization strategies. WSpz vaccines employ *Plasmodium* spz, either attenuated or administered under the cover of a drug that eliminates the parasite’s blood stages as they egress from the liver, to induce strong immune responses against the PE parasite stages, preventing the establishment of a blood stage infection and, therefore, blocking the cycle of transmission. WSpz vaccination approaches include radiation-attenuated spz (RAS), whose growth arrests at an early liver stage^11^, genetically attenuated parasites (GAPs), whose hepatic development is blocked at either an early (EA-GAP)^12^ or at a late (LA-GAP)^13^ stage and chemoprophylaxis and spz (CPS), which employ fully-infectious spz that complete liver stage development and are eliminated by a schizonticidal drug upon the first round of intraerythrocytic schizogony^14^.

Some pre-clinical studies suggested that WSpz formulations relying on immunizing parasites with longer hepatic development provide superior immunity and higher protective efficacy than the ones based on parasites with shorter development in the liver, potentially due to increased parasite biomass, leading to the recognition of a wider and more diverse antigenic repertoire by the host’s immune system. Specifically, immunization of mice with a *P. yoelii* (*Py*) LA-GAP has been shown to elicit higher protection than its EA-GAP and RAS counterparts^15^, and immunization of mice with *Pb* CPS employing azithromycin was reported to display stronger protective immunity than *Pb* RAS^16^. The superiority of LA-GAP relative to EA-GAP has now been confirmed in the clinic with the demonstration that *Pf*Δ*mei2* (GA2), induced significantly higher protective immune responses in humans immunized thrice by the bites of 50 infected mosquitoes than those achieved with *Pf*Δ*b9*Δ*slarp* (GA1)^17^. However, a range of host and environmental factors, as well as vaccine-intrinsic features, have been suggested to influence vaccine immunogenicity and/ or efficacy (reviewed in ref. ^18^). The latter include the type of vaccine, the use of adjuvants, the route of administration, the number and timing of immunizations, and the vaccine dosage^18^. A deeper understanding of all these aspects is required for further improvement of vaccine efficacy and its eventual implementation in the field.

We employed a rodent model of malaria to assess the impact of the immunization dosage on the protective efficacy conferred by immunization with one prime and two boosts (P2B) of *P. berghei* (*Pb*)-based surrogates of the different WSpz formulations, including (i) genetically attenuated WSpz that arrest early in the liver (EA-GAP; *Pb*Δ*b9*Δ*slarp*^19^), (ii) radiation-attenuated spz (RAS^20^), (iii) a newly generated genetically attenuated WSpz that arrest late in the liver (LA-GAP; *Pb*Δ*mei2*Δ*lisp2*), and (iv) non-attenuated wild-type spz administered under the cover of chloroquine (CPS^21^). Our results indicate that while the protection afforded by RAS, LA-GAP and CPS is proportional to the immunization dosage employed, the protective efficacy of EA-GAP does not reach 100% at any regimen tested and decreases markedly above a certain dosage threshold. We further showed that the frequency of effector memory CD8+ T cells with an intermediate phenotype between liver-resident and short-lived effector cells is lower for high-dosage EA-GAP than for high-dosage RAS WSpz immunization. This suggests that effector CD8+ T cells elicited by EA-GAP WSpz immunization present limited developmental plasticity, which may negatively impact the functional versatility of memory cells and, thus, protective immunity^22^. Our findings do not support the prioritization of EA-GAP over RAS, LA-GAP and CPS for malaria vaccination and shed new light on the immune landscape ensuing immunization with different WSpz formulations, guiding their future optimization.

## Results

### Generation, genotyping, and phenotyping of the *Pb*Δ*mei2*Δ*lisp2* parasite line

A previous study identified a role for meiosis inhibited 2 (MEI2), a member of a family of RNA-binding proteins containing an RNA recognition motif^23^, during late liver stage development of *Pb*^13^. However, occasional breakthrough blood stage infections upon inoculation with high number (200 K) of *Pb*Δ*mei2* spz were observed^13^. In addition to *mei2*, a single gene deletion of the liver-specific protein 2 (*lisp2*), a gene expressed on the mid-to-late liver stage parasitophorous vacuole membrane, was reported to lead to late liver stage developmental arrest in *Pb*^24^. We employed the available *Pb*Δ*mei2* (2834cl2m1cl1) parasite line^13^ to create the double gene-deletion mutant *Pb*Δ*mei2*Δ*lisp2*, as was previously reported for *Py*^24^ (Supplementary Fig. 1a). Three independent clones of mCherry-Luc_con_-expressing *Pb*Δ*mei2*Δ*lisp2* were generated and clone 3 (2900cl3) was selected for further analysis (Supplementary Fig. 1b). Our results show that the WT and *Pb*Δ*mei2*Δ*lisp2* parasite lines present comparable numbers of spz (Supplementary Fig. 1c), indicating that the deletion of *lisp2* did not significantly impact the mosquito infectivity of the resulting transgenic parasites. Furthermore, mice injected with up to 3 ×10^5^ spz of *Pb*Δ*mei2*Δ*lisp2* did not develop blood stage infections indicating complete liver stage growth arrest of *Pb*Δ*mei2*Δ*lisp2* (Supplementary Fig. 1d). Live imaging of mCherry-expressing parasites further revealed no differences between the size of WT and *Pb*Δ*mei2*Δ*lisp2* exoerythrocytic forms (EEFs) in cultured hepatocytes at 24 h post infection (hpi). However, at 48 and 72 hpi *Pb*Δ*mei2*Δ*lisp2* liver stages were significantly larger than WT parasites, which is most evident at the 72 h time-point (1399 µm^2^ for *Pb*Δ*mei2*Δ*lisp2* compared to 603 µm^2^ for WT parasites; Supplementary Fig. 1e, f). Immunofluorescence microscopy analysis additionally revealed that *Pb*Δ*mei2*Δ*lisp2* liver stage schizonts expressed merozoite surface protein 1 (MSP1) and apical membrane antigen 1 (AMA1), two proteins that are expressed by early and late *Pb* blood stage schizonts, respectively^25,26^, at 72 hpi (Supplementary Fig. 1g). These observations confirm that *Pb*Δ*mei2*Δ*lisp2* develops to late-stage hepatic parasites, including the expression of antigens associated with a late phase of schizont development.

### Different WSpz formulations present distinct liver stage development profiles

**Fig. 1:**
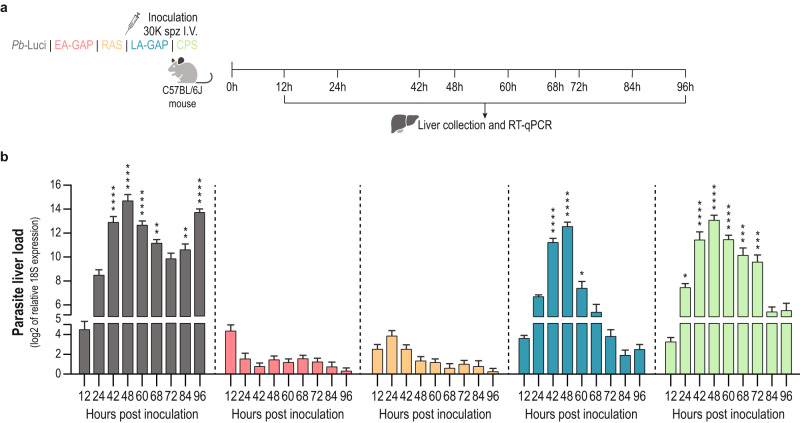
Parasite liver load of C57BL/6J mice inoculated with different *Pb*-based surrogates of WSpz formulations. **a** Study protocol. C57BL/6 J mice were inoculated intravenously (I.V.) with 30 K spz of *Pb*-Luci, EA-GAP, RAS, LA-GAP, or *Pb*-Luci administered under a chemoprotective chloroquine regimen, and euthanized at the indicated time-points post inoculation for liver collection and RT-qPCR analysis. **b** Parasite load in mouse livers collected at the indicated time-points post spz inoculation (*n* = 6–9 mice per time-point from 2-3 independent experiments). For each time-point, data are expressed as mean ± SD and were compared using the Kruskal–Wallis test with Dunn’s multiple comparison post-test (*P < 0.05; **P < 0.01; ***P < 0.001; ****P < 0.0001).

Evidence has been presented that the extent of parasite development in the liver is an important determinant of the protective efficacy of different WSpz formulations^15–17^. In this study we compared the protective efficacy afforded by immunization of mice with four different *Pb*-based WSpz formulations: EA-GAP (*Pb*Δ*b9*Δ*slarp*^19^), RAS^20^, LA-GAP (*Pb*Δ*mei2*Δ*lisp2*) and CPS^21^. In order to assess the development profiles of the *Pb* parasites employed as surrogates of WSpz vaccines we first determined the parasite load of livers of C57BL/6J mice injected intravenously (I.V.) with 30 K spz of each formulation. As a control, mice were infected with 30 K luciferase-expressing *Pb* spz (hereafter referred to as *Pb*-Luci) without chloroquine treatment. Mouse livers were collected at specific time-points following spz inoculation, spanning the entire period of *Pb* hepatic development (12–60 h post inoculation - hpi), as well as the initial stages of erythrocytic infection (68–96 hpi)^4^, and the parasite liver load was determined by quantitative reverse transcription PCR (RT-qPCR) (Fig. 1a).

As expected, the parasite biomass in mice infected with *Pb*-Luci linearly increased up to 48 hpi, corresponding to the period of growth and multiplication of the parasites into mature liver stages, followed by a decrease in liver load between 60 and 72 hpi, presumably as a result of the completion of the parasite’s hepatic development and the release of liver merozoites. An increase in parasite biomass is observed between 84 and 96 hpi, likely due to the presence of *Pb* blood stages in the liver sinusoids (Fig. 1b). The parasite biomass in mice inoculated with EA-GAP and RAS WSpz is markedly lower than that of mice inoculated with *Pb*-Luci at all time-points, which is consistent with the early growth-arrest of those parasites in the liver^19,27^. However, small differences exist between these two WSpz formulations; while the parasite biomass in EA-GAP-inoculated mice is highest at 12 hpi, RAS-inoculated mice reach an equivalent load at 24 hpi (Fig. 1b), indicating that liver growth of EA-GAP arrests earlier than RAS. The parasite biomass in mice inoculated with LA-GAPs or CPS WSpz formulations displays a pattern similar to that of *Pb*-Luci up to 48 hpi, indicating wild-type-like growth of these parasites into late liver stages. However, parasite biomass at later time-points decreases more rapidly in LA-GAP WSpz- than in CPS WSpz-inoculated mice, presumably due to the former parasite’s developmental arrest and eventual elimination from the liver. Conversely, in CPS WSpz-inoculated mice, hepatic parasites develop into fully mature schizonts that release liver merozoites, which are eliminated by chloroquine (Fig. 1b).

### Protective efficacy of the different WSpz formulations is dependent on the immunization dosage

**Fig. 2:**
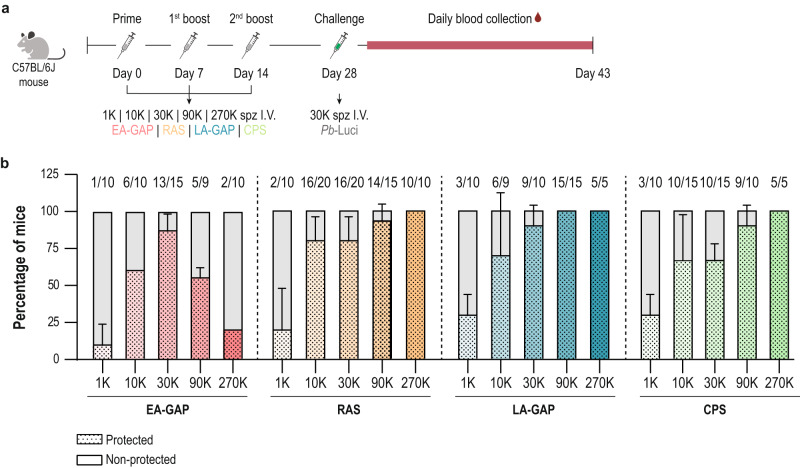
Sterile protection of C57BL/6J mice immunized with different dosages of the various WSpz formulations following a P2B vaccination regimen. **a** Study protocol. C57BL/6J mice were immunized following a P2B immunization regimen administered via I.V. injection of 1, 10, 30, 90 and 270 K of the EA-GAP, RAS, LA-GAP and CPS WSpz formulations. Non-immunized and immunized mice were infected/challenged on day 28 with 30 K spz of *Pb-*Luci. Blood was collected daily for a period of 15 days and analyzed by a bioluminescence assay. **b** Percentage of protected (in colored and dotted pattern bars) and non-protected (in gray bars) mice for each immunization dosage and vaccination approach (*n* = 5–20 mice per immunization dosage from 1–4 independent experiments). Sterile protection is defined as the absence of blood stage parasites for up to 15 days following the infectious challenge. Numbers above the bars indicate number of protected/total number of challenged mice. For each immunization dosage, the percentage of protected mice are expressed as mean ± SD.

WSpz immunization has resulted in variable levels of protective efficacy, both in rodent malaria models and in humans, depending on factors such as the route of administration, and immunization dosage and schedule^28–30^. Since previous studies have underscored the need for optimization of the WSpz dosage required to achieve robust and sterile protection^30^, we sought to assess the impact of the immunization dosage on the protective efficacy of the four different WSpz formulations. To this end, C57BL/6J mice were immunized following a P2B regimen with weekly intervals, an immunization regimen previously demonstrated to induce highly protective immune responses in mouse malaria models^31,32^, employing five distinct WSpz dosages (1, 10, 30, 90, and 270 K). On day 28, two weeks after the last immunization, mice were challenged with 30 K fully infectious *Pb*-Luci spz, and relative parasitaemia was monitored for at least 15 days thereafter by bioluminescence^33^. Control mice (hereafter referred to as Challenge controls) infected with 30 K *Pb*-Luci spz at the time of challenge (Fig. 2a) developed a blood stage infection with a 3-day pre-patent period (Supplementary Fig. 2a).

The protective efficacy induced by the different dosages of the four WSpz formulations was assessed by induction of sterile protection, as defined by the absence of detectable blood stage infection until 15 days after challenge. The percentage of protected mice followed a similar, dosage-dependent increase up to a dosage of 30 K of all WSpz formulations (Fig. 2b). The dosage-dependent increase in protection is further observed for RAS, LA-GAP and CPS at WSpz dosages above 30 K. Between 90 and 100% of the immunized mice were sterile protected at immunization dosages of 90 and 270 K spz. Remarkably, however, not only was sterile protection not observed for all mice immunized with any given dosage of EA-GAP WSpz, but also immunization with 90 and 270 K WSpz dosages of this formulation led to a 1,6- and 4,3-fold reduction in sterile protection levels, respectively, relative to those observed for immunization with 30 K WSpz (Fig. 2b).

In addition to sterile protection, protective immunity was assessed by determining the pre-patent period in the mice that developed a blood infection after challenge with 30 K *Pb*-Luci spz. A longer pre-patent period has been correlated with a reduction in the inoculum of parasites emerging from the liver into the blood^34^. Overall, the pre-patency profile of the non-sterile protected mice followed a trend similar to that observed for sterile protection, with the pre-patent period of mice immunized with RAS, LA-GAP and CPS increasing with immunization dosage. In contrast, the maximum delay in patency for EA-GAP WSpz-immunized mice was observed at a 30 K dosage and decreased at the higher dosages of 90 and 270 K WSpz (Supplementary Fig. 2a). *Pb* ANKA-infected C57BL/6J mice constitute a well-established model of experimental cerebral malaria (ECM), enabling the assessment of protection against ECM induced by different immunization approaches. Overall, protection against the development of ECM follows a pattern similar to that observed for sterile protection afforded by the different dosages and WSpz formulations (Supplementary Fig. 2b and Fig. 2b). Nevertheless, 10 K or higher dosages of CPS WSpz appear to offer the highest protection against this severe syndrome. Additionally, while all non-sterile protected mice immunized with 270 K EA-GAP developed ECM^35^, only 20 ± 28.3% of the non-sterile protected mice immunized with 1 K EA-GAP succumbed to ECM (Supplementary Fig. 2b). Thus, high dosage immunization with EA-GAP affords low levels of not only sterile protection and delayed patency, but also protection against severe disease.

Overall, our results show that, whereas sterile protection was not observed for all mice immunized with any given dosage of EA-GAP WSpz, 100% of the animals immunized with 270 K RAS, 90 and 270 K LA-GAP, and 270 K CPS were sterile protected against challenge. Moreover, while protection conferred by RAS, LA-GAP and CPS correlates with WSpz dosages from 1 to 270 K, EA-GAP WSpz dosages higher than 30 K result in a decline in protective efficacy. Somewhat surprisingly, we also found that protection induced by immunization with RAS WSpz was comparable to that afforded by LA-GAP and CPS WSpz, indicating the absence of a strong correlation between protective immunity and the extent of parasite development and biomass in the liver.

### Low protection following high-dosage EA-GAP immunization is not associated with evidence for liver damage or reduced magnitude of the immune responses

**Fig. 3:**
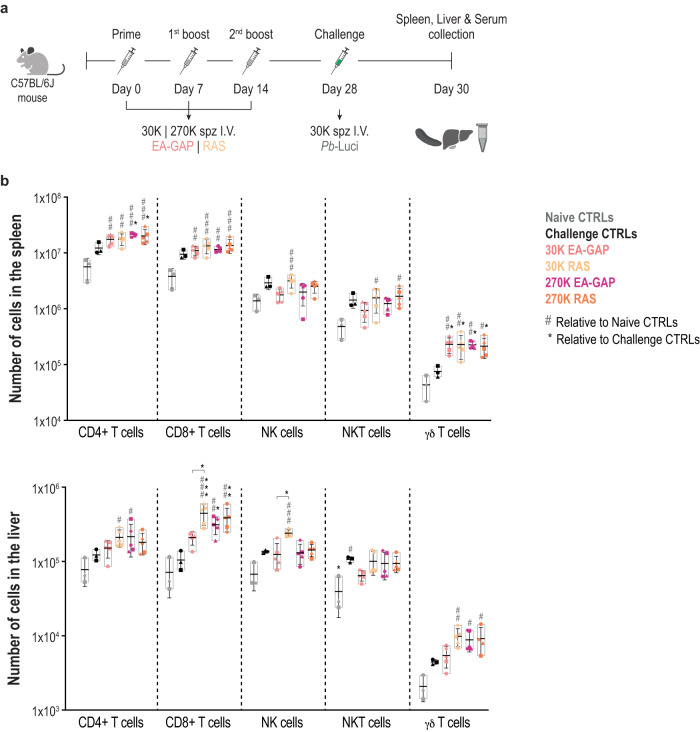
Immune population analyses of the spleens and livers of C57BL/6J mice immunized with either 30 or 270 K of EA-GAP or RAS WSpz following a P2B immunization regimen. **a** Study protocol. C57BL/6J mice were immunized by I.V. injection of 30 or 270 K EA-GAP or RAS WSpz. Non-immunized and immunized mice were subsequently infected/challenged on day 28 with 30 K spz of *Pb-*Luci. Forty-eight hours post challenge (day 30), mice were euthanized for spleen, liver, and serum collections. **b** Number of CD4+ T, CD8+ T, NK, NKT and *γδ* T cells in the spleen (top) and liver (bottom) of naïve and challenge controls, as well as of immunized mice (*n* = 3–5 per group). Symbols represent the individual values of each mouse and floating bars indicate the mean ± SD. Statistically significant differences relative to naïve control mice (#), relative to challenge controls (* above the bars) or between experimental groups (* above the lines) were assessed through the Kruskal–Wallis test with Dunn’s multiple comparison post-test (*^,#^*P* < 0.05; **^,##^*P* < 0.01; ***^,###^*P* < 0.001; ****^,####^*P* < 0.0001).

The low protection levels observed following immunization with EA-GAP WSpz dosages higher than 30 K prompted us to investigate possible causes behind this lower protective efficacy compared to the other WSpz formulations. A previous in vitro study showed that hepatocytes infected with another early-arresting parasite, *Pb*Δ*p36p*, presented higher levels of apoptosis than RAS-infected cells^36^. Similarly to the EA-GAP employed in the present study, *Pb*Δ*p36p* fails to maintain a parasitophorous vacuole and arrests soon after hepatocyte invasion^36^. Thus, we hypothesized that increased levels of apoptosis of infected hepatocytes following three immunizations with high EA-GAP dosages might compromise the ability of the booster parasites, to establish protective immune responses. To investigate the hepatocellular damage resulting from three EA-GAP WSpz immunizations, histopathological analyses were performed on livers from mice immunized with 270 K EA-GAP WSpz, collected 48 h after the challenge with *Pb*-Luci parasites. To limit the number of animals used in this study, and since RAS immunization is considered the gold-standard of WSpz immunization^7^, mice immunized with 270 K RAS WSpz were employed as controls in these experiments. In addition, to further control for the potential influence of the load of immunizing parasites, two additional groups of mice, immunized with 30 K EA-GAP or RAS WSpz were included in these analyses (Fig. 3a). Livers of all immunized animals presented infiltrates indicative of a chronic (longer than 2 days) inflammation (Supplementary Fig. 3a), which is absent from non-immunized, challenged mice, and thus likely results from the immunization rather than from the challenge. Nonetheless, no significant histopathological differences or distinct patterns of microscopically detectable hepatocellular damage were found between mice immunized with either dosage of the EA-GAP and RAS WSpz (Supplementary Fig. 3a). Additionally, we measured known biochemical parameters associated with liver function^37^ in serum collected from these immunized mice. In agreement with the histopathological analysis (Supplementary Fig. 3a), no significant differences in the serum levels of total bilirubin and glucose were found between the four groups of immunized mice. In addition, the levels of aspartate aminotransferase (AST) and alanine aminotransferase (ALT) are comparable between mice immunized with the same dosage of either WSpz formulation (Supplementary Fig. 3b). Collectively, our data indicate that the low protection conferred by high dosage EA-GAP immunization is not associated with evidence for WSpz-induced liver injury or disfunction.

Immunization with WSpz formulations induces strong immune responses against the pre-erythrocytic parasite stages^7^. RT-qPCR analysis of immunized mouse livers collected 2 days after challenge (Fig. 3a) revealed that the *Pb*-Luci parasite biomass was higher in EA-GAP WSpz- than in RAS WSpz-immunized mice, particularly for animals immunized with 270 K WSpz (Supplementary Fig. 3c), in agreement with the sterile protection results shown in Fig. 2b. A standard flow cytometry hierarchical gating strategy (Supplementary Fig. 3d) was employed to assess potential immune factors involved in the low protective efficacy conferred by immunization with high dosage EA-GAP WSpz. To this end, lymphocyte populations isolated from the spleens, as a proxy for systemic immune responses, and livers of mice immunized with 30 and 270 K EA-GAP WSpz 48 h after challenge were compared with those from spleens and livers of mice immunized with 30 and 270 K RAS WSpz (Fig. 3a). This analysis was performed after challenge because that enables elucidating the overall immune response in a vaccination/infection setting. In addition, we ensured the rigor of this analysis by the use of appropriate control groups, including naïve and non-immunized mice subjected to the same challenge, providing information on the contribution of the mobilization of immune cells from the challenge alone.

Overall, no significant differences were found in the magnitude of the splenic immune responses between the four groups of immunized mice (Fig. 3b). Nonetheless, it is worth mentioning that CD4+, CD8+ and *γ*δ T cell populations generally increased in immunized mice relative to the naïve or to the challenge controls (Fig. 3b). We further analyzed the same set of immune populations in the livers of EA-GAP WSpz- and RAS WSpz-immunized mice (Fig. 3b). Our results show that, whereas immunization with 30 K EA-GAP WSpz did not lead to significant differences in the numbers of CD4+ T, CD8+ T, NKT, *γ*δ T and NK cells in the liver compared to naïve and challenge controls, immunization with the same dosage of RAS WSpz induced significant increases in the cell numbers of all lymphocyte populations analyzed, except NKT cells (Fig. 3b). This suggests that the EA-GAP and RAS WSpz immunizations induce local immune responses of significantly different magnitudes, which may be related to differences in the extent of liver stage development of either WSpz formulation. On the other hand, mice immunized with 270 K spz of either EA-GAP or RAS WSpz exhibit numbers of CD4+, *γδ* and NK T cells equivalent to those found in the livers of mice immunized with 30 K RAS WSpz. This indicates that a 9-fold increase in immunization dosage with either EA-GAP or RAS WSpz relative to the 30 K RAS WSpz inoculum does not elicit a substantial difference in the magnitude of immune cell responses in the liver (Fig. 3b).

Overall, our analyses did not identify any significant differences in the magnitude of either systemic or liver immune responses that could be associated with the low protection levels observed upon immunization with high dosage EA-GAP WSpz.

### High dosage EA-GAP immunization results in lower frequency of effector CD8+ T cells with an intermediate phenotype between T_RM_ and SLECs

**Fig. 4:**
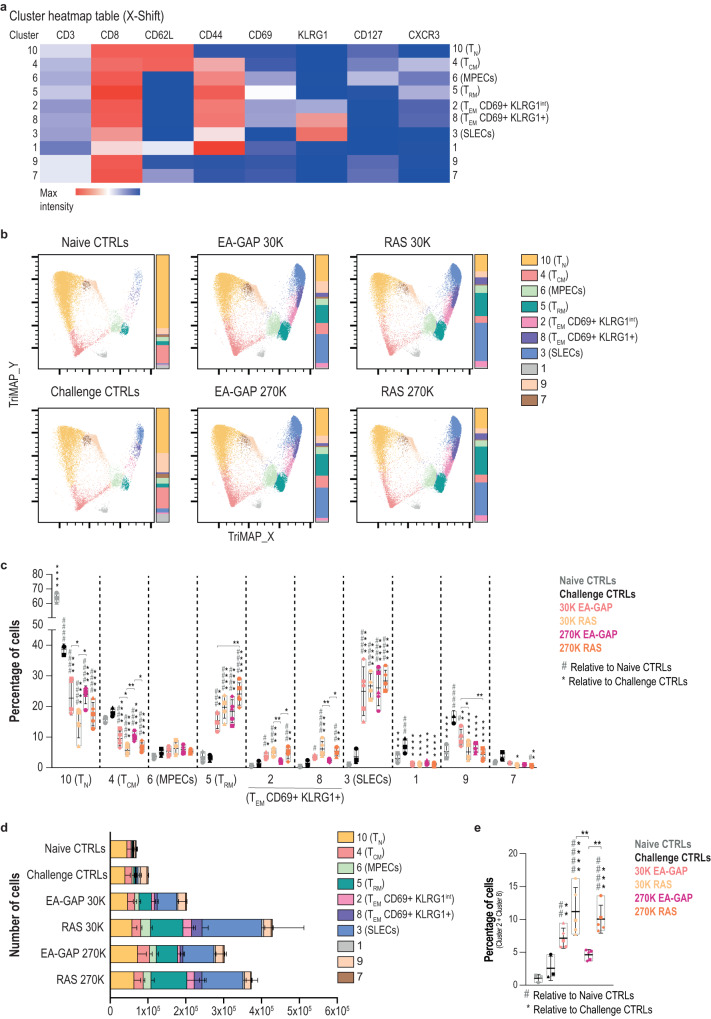
Clustering analysis of the CD8+ T cell pool in the liver of C57BL/6J mice immunized with either 30 or 270 K EA-GAPs or RAS WSpz following a P2B immunization regimen. **a** Heatmap of clusters identified by X-shift analysis of concatenated data from CD8+ T cells of the livers of naïve and challenge control mice, as well as of immunized mice, 48 h post challenge (day 30). T_N_: naïve T cells; T_CM_: central memory T cells; T_EM_: effector/effector memory T cells; T_RM_: resident memory T cells; SLECs: short-lived effector cells; MPECs: memory-precursor effector cells; KLRG1: killer cell lectin-like receptor subfamily G, member 1; CXCR3: C-X-C motif chemokine receptor 3. **b** X-shift clustering analysis of concatenated data from the liver of mice under each condition. Data is presented using TriMap dimensionality reduction. Parts-of-whole bar graphs next to each plot represent the clusters within the CD8+ T cell population of each condition. Frequencies (**c**) or numbers (**d**) of each of the clusters identified in the X-shift analysis in the liver, for each condition. Symbols in **c** represent the individual values of each mouse and floating bars indicate the mean ± SD. Statistically significant differences relative to naïve control mice (#), relative to challenge controls (*above the bars) or between experimental groups (* above the lines) were assessed through the Kruskal–Wallis test with Dunn’s multiple comparison post-test (^*,#^*P* < 0.05; **^,##^*P* < 0.01^; *^**^,###^*P* < 0.001; ****^,####^*P* < 0.0001). Data in **d** are presented as mean ± SD. **e** Combined frequency of clusters 2 and 8, representing total CD8+ T_EM_ CD69+ KLRG1+ T cells within liver leukocytes. Symbols represent the individual values of each mouse and floating bars indicate the mean ± SD. Statistically significant differences relative to naïve control mice (#), relative to challenge controls (*above the bars) or between experimental groups (* above the lines) were assessed through the Kruskal–Wallis test with Dunn’s multiple comparison post-test (*^,#^*P* < 0.05; **^,##^*P* < 0.01; ***^,###^*P* < 0.001; ****^,####^*P* < 0.0001).

CD8+ T cells have been identified as key mediators of protective immunity elicited by different WSpz formulations in rodent models of malaria^38^. Notably, a relationship between antigen dose and functional avidity, memory phenotype and survival of CD8+ T cells has been demonstrated in different models, such as *Leishmania major*, HIV and *Mycobacterium tuberculosis*, with lower dosages providing increased protection, indicating that lower antigen doses lead to more favorable T cell responses (reviewed in ref. ^39^). Hence, we further investigated the composition of the liver CD8+ T cell population applying an unbiased clustering algorithm to our flow cytometry data^40^. To this end, the X-shift algorithm was applied to concatenated datasets consisting of CD8+ T cell populations from the livers of naïve and challenge control mice, as well as from livers of mice immunized with 30 and 270 K EA-GAP or RAS WSpz, collected 48 h post challenge and the clustering analysis was visualized using TriMap dimensionality reduction (Fig. 4a, b). The phenotypic classification of the CD8+ T cell subsets was primarily based on the expression of CD62L (L-selectin), a lymph node homing molecule, and CD44, a molecule involved in cell adhesion, migration, and signaling^41^. Three main populations were identified: i) naïve T cells (T_N_; CD62L+ CD44−, cluster 10); ii) central memory T cells (T_CM_; CD62L+ CD44+, cluster 4); and iii) effector/effector memory T cells (T_EM_; CD62L− CD44+; clusters 2, 3, 5, 6, 8; Fig. 4a). The latter subset has been reported to consist of a quite heterogeneous pool of CD8+ T cells, comprising distinct subpopulations of T_EM_ cells^42^. Our analysis identified these subpopulations as including resident-memory cells (T_RM_; CD69+ KLRG1− CXCR3+, cluster 5), memory-precursor effector cells (MPECs; KLRG1- CD127+, cluster 6) and short-lived effector cells (SLEC; KLRG1+ CD127−, cluster 3), each with distinctive memory potential and functional properties^43^ (Fig. 4a). Interestingly, in addition to previously known cell populations, our X-shift analysis identified two populations of T_EM_ co-expressing CD69 and KLRG1 (CD69+ KLRG1^int^, cluster 2 and CD69+ KLRG1+, cluster 8), which appear to present an intermediate phenotype between T_RM_ and SLECs (clusters 8 and 2 phenotypically closer to SLECs and T_RM_, respectively; Fig. 4a, b).

As expected, a statistically significant increase in the frequency of CD8+ SLECs and T_RM_ was observed in all four groups of immunized mice relative to both naïve and challenge controls, which is also observed in terms of cell numbers except for the mice immunized with 30 K EA-GAP (Fig. 4c, d, Supplementary Fig. 4). Of note, the numbers of CD8+ T_RM_ are more comparable between mice immunized with the different dosages of RAS WSpz than between mice immunized with the same dosage of the two WSpz formulations (Fig. 4d, Supplementary Fig. 4). This indicates that RAS immunization induces higher levels of CD8+ T_RM_ than EA-GAP immunization, irrespective of the immunization dosage. Likewise, mice immunized with either EA-GAP WSpz dosage present similar levels of hepatic CD8+ T clusters corresponding to T_N_ and T_CM_, which were higher than those of RAS-immunized mice (Fig. 4c). Of particular interest, we observed an increase in the frequency of clusters 2 and 8 in all immunized mice, except for those immunized with 270 K EA-GAP WSpz, which showed significantly lower frequencies of those clusters than either group of RAS WSpz-immunized mice. A similar trend for lower frequencies of clusters 2 and 8 was observed in mice immunized with 270 K EA-GAP WSpz in comparison with those immunized with 30 K of this formulation, although it did not reach statistical significance (Fig. 4c). The results of a joint analysis of clusters 2 and 8 were similar to those obtained for the analyses of either individual cluster, including the statistically significant difference between the mice in the 270 K EA-GAP WSpz-immunized group and both groups of RAS WSpz-immunized mice (Fig. 4e).

In conclusion, our results indicate that, contrarily to RAS immunization, immunization with a high dosage of EA-GAP WSpz did not induce the expansion of an intermediate T_EM_ population expressing markers of both effector and liver-resident memory CD8+ T cells, which may be related to the relatively low levels of protective efficacy observed with the latter immunization regimen.

## Discussion

It has long been assumed that the protective efficacy of WSpz malaria vaccines depends on the hepatic antigen load attained following administration of immunizing parasites with distinct extents of liver development^15,16^. This assumption was recently strengthened by the results of a clinical study showing that immunization with LA-GAP conferred higher protection against controlled human malaria infection than its EA-GAP WSpz counterpart^17^. However, whether protection depends exclusively on the biomass of liver parasites or is also impacted by other factors, such as WSpz dosage or WSpz formulation-intrinsic features, needs to be further assessed. Thus, we employed a rodent model of malaria and a P2B immunization regimen to address the impact of WSpz dosage on the protective efficacy of different types of WSpz formulations. Our results unequivocally show that whereas mice immunized with increasing dosages of RAS, LA-GAP and CPS WSpz exhibit similar and progressively higher levels of sterile protection against challenge with fully-infectious spz, immunization with high dosages (90 K and 270 K WSpz) of EA-GAP is associated with a decrease in protection against a similar challenge. Moreover, none of the five dosages of EA-GAP WSpz employed in this study conveyed sterile protection to 100% of the immunized animals.

The similar levels of sterile protection following immunization with RAS and LA-GAP may be somewhat unexpected, considering previous reports indicating that the LA-GAP *Py*Δ*fabb/f* conferred significantly higher protection against infectious challenge than both *Py* RAS and the EA-GAP *Py*Δ*sap1*^15^. This apparent discrepancy may be explained by the differences not only in the immunization regimen and time-point of challenge, but also in the parasite species employed the two studies. In fact, previous studies have shown that *Py*-based WSpz immunization yields higher protection levels than its *Pb* counterpart, particularly when employing LA-GAPs (reviewed in^44^). On the other hand, CPS WSpz vaccination has previously demonstrated superior protective efficacy at a much lower dose compared to RAS^45^, which is not observed in the current study. However, with the benefit of hindsight, and as noted by the authors of the previous manuscript, it is now apparent that the administration of 2.7 ×10^6^ RAS into the volunteers was not optimal, given the low efficacy obtained following the inoculation of 1.8 ×10^6^ parasites in Tanzanian volunteers in an independent clinical trial^30^.

The liver stage of *Plasmodium* infection, potentially due to its short duration and the relatively small proportion of infected hepatocytes, does not cause significant hepatic damage or pathology^46^. Nonetheless, the host’s response to infection can result in liver injury due to the influx of immune cells, which may lead to hepatic inflammation and tissue damage^47^. Furthermore, it has been reported that hepatic cells infected in vitro with a *Pb* mutant lacking expression of the P36p protein exhibit a significantly higher degree of apoptosis than hepatocytes infected with *Pb* RAS under the same experimental conditions^36^. P36p-deficient parasites are similar to the EA-GAP employed in the present study in that both are able to invade hepatocytes, but arrest development shortly thereafter due to their inability to maintain an intact parasitophorous vacuole^48^. Based on these observations, we hypothesized that the delivery of a high EA-GAP WSpz inoculum may enhance hepatocytic apoptosis, potentially compromising the ability of the WSpz in the subsequent boost immunizations to productively invade the hepatocytes and elicit protective immune responses. However, we found that mice immunized with 30 or 270 K EA-GAP or RAS WSpz present similar signs of immunization-related chronic inflammation, and no significant differences in apoptosis and liver function. This suggests that the low protection observed following immunization with high dosage EA-GAP WSpz does not result from liver injury or disfunction. However, it should be noted that our analysis of liver samples was conducted 16 days after the final immunization, which may have allowed enough time for the liver to recover^49^, potentially leading to an oversight of hepatic injury.

Previous WSpz vaccination studies employing the Sanaria® PfSPZ Vaccine (Sanaria Inc., Rockville, MD), composed of aseptically purified, cryopreserved *Pf* RAS WSpz, have shown that increasing the number of WSpz per dose can enhance the vaccine’s protective efficacy^11,50–52^. However, in a study of this vaccine with Tanzanian volunteers, escalating the WSpz dose led to a significant reduction in protective efficacy^30^. It was hypothesized that a phenomenon of high-dose tolerance/suppression of T cells, in which supra-optimal engagement of the T-cell receptor leads to reduced proliferation, cytokine secretion and/or apoptosis of activated cells^53,54^, could play a role in the observed decrease of the protection conferred by the vaccine. However, the analysis of immune responses using lymphocytes present in circulation, rather than liver-associated immunity^55^, may have hampered the identification of correlates for that loss of protection. Seminal studies performed with viral and bacterial vaccines suggested that high antigen doses were required to elicit protection through the induction of sufficient antibody titers (reviewed in ref. ^56^). However, more recent research on vaccination against pathogens that cause persistent or chronic intracellular infections has demonstrated the importance of priming T cell responses by immunization, in addition to humoral immunity (reviewed in ref. ^39^). In some of these studies, vaccination with lower antigen doses was associated with more favorable CD4+ and CD8+ T cell responses and increased protective efficacy, in both animal models and humans^57–59^.

CD8+ T cells have been implicated as key effector cells that play a central role in protection against *Plasmodium* infection following immunization^38^. The first evidence of their importance in the protective immunity elicited by WSpz immunization was demonstrated in mice immunized with RAS WSpz^60^. It has been reported that high antigen dosages can have a detrimental effect on CD8+ T cell responses and were associated with a decreased quality of responding T cells, including lower functional avidity^59^, loss of memory phenotype^61,62^ and deletion by exhaustion caused by the activation of a large fraction of available T cell precursors^59,63^. Our unbiased clustering analysis of CD8+ T cells identified three CD69+ CD8+ T cell populations that could be distinguished based on the higher (cluster 8), intermediate (cluster 2) or lower (cluster 5) expression levels of KLRG1, a marker of cells with an effector phenotype^64^. In line with previous findings from our group^31^, we observed an immunization-associated expansion of clusters 2 and 8, which appear to present an intermediate phenotype between CD69+ KLRG1− T_RM_ and CD69- KLRG1+ SLECs. However, the frequency of those populations in mice immunized with 270 K EA-GAP WSpz was clearly different from that observed in the remaining groups of immunized mice. Specifically, and contrary to the latter, their frequency in the former (i) did not significantly increase relative to control mice, and (ii) was significantly lower than in mice immunized with either dosage of RAS WSpz. It has been reported that, in addition to KLRG1- MPECs, CD8+ KLRG1+ T cells can also differentiate into all memory T cell lineages, including T_RM_ (named exKLRG1 memory cells), fostering the pool of memory cells with diverse functional capabilities, and ensuring long-lasting protective immunity^22^. This study also showed that exKLRG1 memory cells presented high cytotoxic and proliferative capacity, which contributed not only to enhanced anti-influenza but also anti-tumor immunity^22^. Therefore, the absence of expansion of clusters 2 and 8 of CD8+ KLRG1+ CD69+ T cells following immunization with the poorly protective high dosage of EA-GAP WSpz suggests that the developmental plasticity of KLRG1+ effector CD8+ T cells induced by this immunization regimen is limited. This possibility, and its potential negative impact on the functional versatility of memory cells and, thus, protective immunity, is currently being investigated.

To the best of our knowledge, this is the first report of a detailed side-by-side analysis of the dosage-dependent protective efficacy of four WSpz formulations. We show that a dosage-dependent protective efficacy of immunization is observed for immunization with up to at least 270 K RAS, LA-GAP and CPS WSpz. Conversely, protection afforded by EA-GAP peaks at a dosage between 30 and 90 K WSpz, albeit without reaching 100% sterile protection, and markedly decreases thereafter. This may be due to a decrease in the developmental plasticity of effector CD8+ T cells, which negatively impacts the ensuing memory responses. These observations, together with those from a previous clinical trial demonstrating the higher efficacy of LA-GAP over the EA-GAP^17^, raise concerns regarding the applicability of early-arresting parasites for WSpz vaccination against malaria. Nonetheless, since a decrease in protection upon an increase in PfSPZ Vaccine dosage has been observed in the clinic^30^, a maximum threshold of WSpz dose may also exist for effective immunization with RAS, LA-GAP and CPS, albeit at a higher dosage than for EA-GAP. Such a possibility should be taken into consideration when fine-tuning WSpz doses for human vaccination. Overall, our findings enhance our understanding of the complexity of the protective immune responses elicited by WSpz immunization and can contribute to guiding the future optimization of vaccination regimens.

## Methods

### Mice

Animal experiments were performed at the rodent facilities of Instituto de Medicina Molecular João Lobo Antunes (iMM JLA, Lisbon, Portugal) and Leiden University Medical Center, which are licensed under the European Directive 2010/63/EU on the Protection of Animals used for Experimental and Other Scientific Purposes. Female OF1, male BALB/c and C57BL/6J mice, aged six to nine weeks, were purchased from Charles River Laboratories (Leiden, Netherlands or Lyon, France) and housed under specific pathogen-free (SPF) conditions. Mice were kept under a 12 h light/dark period at a temperature of 25 °C and 40–70% relative humidity. Filtered tap water and *γ*-irradiated pelleted diet were provided *ad libitum*. All experimental procedures were performed in strict compliance with Federation of European Laboratory Animal Science Associations (FELASA) guidelines and approved by the Animal Experiments Committee of Leiden University Medical Center (DEC 12042 and 14207) and by the iMM JLA’s animal ethics committee (ORBEA-iMM).

### Parasite lines and mosquito infection

*Anopheles stephensi* (*A. stephensi*) mosquitoes were bred and maintained in controlled conditions in the Insectary Facility of iMM-JLA, under 12 h light/dark cycles, 21 °C and 80% humidity. For the generation of the double gene-deletion mutant, the selectable marker (SM)-free single gene-deletion mutant *Pb*Δ*mei2* (2834cl2m1cl1, mutant RMgm-4937; http://www.pberghei.eu)^13^ was employed which was previously generated upon transfection of a parental WT line (1868cl1). The WT (1868cl1, mutant RMgm-1320; www.pberghei.eu) line (*Pb*ANKA-mCherry_hsp70_+Luc_eef1α_)^65^ contains the fusion *mCherry* gene under control of the *heat shock protein* (*hsp70*) promoter and the *luciferase* gene under control of the constitutive *eef1α* promoter integrated into the neutral *230p* gene locus (PBANKA_0306000). For immunizations and challenge experiments, female *A. stephensi* mosquitoes were infected with the following parasite lines: (i) GFP/luciferase-expressing *Pb* ANKA parasite line (GFP-Luc_CON_; 676m1cl1, mutant RMgm-29; www.pberghei.eu)^66^, referred to as *Pb*-Luci, (ii) *Pb*Δ*b9*Δ*slarp* parasites (1844cl1, mutant RMgm-1141; www.pberghei.eu), in which the expression of B9 and SLARP proteins has been abrogated^19^, and (iii) *Pb*Δ*mei2*Δ*lisp2* parasites (2900cl3, mutant RMgm-4937; www.pberghei.eu), which are genetically attenuated by the deletion of the *mei2* and *lisp2* genes.

### Generation and genotyping of the double gene-deletion mutant *Pb*Δ*mei2*Δ*lisp2*

Previously generated *Pb*Δ*mei2* parasites^13^ and the gene deletion construct pL1462^65^ were used to delete the *lisp2* gene (PBANKA_1003000). pL1462, which contains the dihydrofolate reductase gene of *Toxoplasma gondii* (*tgdhfr*) flanked by the *dhfr* of *Pb* (*pbdhfr*) promoter region and the 3’-UTR of *pbdhfr*, replaces the complete *lisp2* open reading frame (ORF) by the *tgdhfr* SM cassette through double cross-over homologous recombination (Supplementary Fig. 1a). Transfection with linearized constructs, positive selection of transfected parasites with pyrimethamine and cloning of selected parasites were performed as described in ref. ^67^. Clonal expansion resulted in three independent clones of line 2900, for which hybridization of pulsed field gel (PFG)-separated chromosomes with the 3’-UTR *pbdhfr* probe recognized the integrated construct on chromosome 10, the endogenous *pbdhfr* gene located on chromosome 7, and the reporter mCherry-Luc_con_ construct on chromosome 3 (Supplementary Fig. 1b). The correct integration of the construct pL1462 in the parasite line *Pb*Δ*mei2*, as shown by the presence of the *tgdhfr* SM and 3’ integration of *lisp2*, as well as the absence of the *lisp2* ORF relative to the WT line, was confirmed through genotype analysis by diagnostic PCR analysis of the 2900cl3 (Supplementary Fig. 1b)^67^. PCR primers used to confirm correct integration of the constructs are listed in Supplementary Table 1.

### Determination of prepatent period after infection

Twenty-one days post infectious blood meal, spz were obtained by dissection of salivary glands from WT- and *Pb*Δ*mei2*Δ*lisp2-*infected *A. stephensi* mosquitoes. Mosquito salivary glands were kept on ice in RPMI culture medium, homogenized with a pestle to release the spz and filtered (40 µm Falcon, Corning, Amsterdam, NL). Free spz were subsequently counted in a Bürker counting chamber using phase-contrast microscopy. C57BL/6J mice were anaesthetized using isoflurane (Steve) and defined dosages of WT and *Pb*Δ*mei2*Δ*lisp2* spz were administered via retro-orbital I.V. Mice were subsequently monitored for blood stage infections by Giemsa-stained blood smears made from 4 to 30 days post-infection. Mice were considered infected when a parasitemia of 0.5-2% was observed.

### In vitro infection of a human hepatoma cell line with *Pb*Δ*mei2*Δ*lisp2* sporozoites

The human hepatoma cell line Huh7 was cultured in RPMI 1640 medium supplemented with 10% (v/v) fetal bovine serum (FBS), 2% (v/v) Penicillin/Streptomycin and 1% (v/v) GlutaMAX (Invitrogen) and maintained at 37 °C with 5% CO_2_. For live imaging and immunofluorescence analyses, cells were seeded (5 ×10^4^ per well) on glass coverslips in 24-well plates and infected 24 h later by adding 5 ×10^4^ freshly dissected spz. At 24, 48 and 72 hpi, nuclei were stained with Hoechst 33342 (Sigma) at a final concentration of 10 µM and live imaging of mCherry-expressing parasites and EEF size were measured using Leica LAS X software by determining the area of the parasite at its greatest circumference using the mCherry-positive area (µm^2^). The following exposure times were used: Alexa 488/FITC: 0.7 s; mCherry: 0.7 s and Hoechst 0.136 s (1x gain). For immunofluorescence analysis, cells were fixed with 4% (v/v) PFA in PBS for 30 min at room temperature (RT) at the indicated time-points. Cells were then permeabilized with 1% Triton X-100 in PBS for 30 min at RT. Parasites were stained with rabbit anti-*Py* MSP1 (1:200 dilution)^68^ and rat anti-*Pf* AMA1 (1:200 dilution)^25^ in 10% fetal calf serum overnight at 4 °C, followed by three washes with PBS at RT. Cells were then incubated with secondary conjugated antibodies anti-rabbit IgG Alexa Fluor^®^ 488 (Invitrogen; 1:200 dilution) or anti-rat IgG FITC (Thermofisher; 1:200 dilution) in the presence of Hoechst 33342 (Sigma) for nuclei staining. Fixed cells were covered with 1–2 drops of the anti-fading agent Vectashield, and a coverslip was placed onto the cells. Stained cells were analyzed by fluorescence using a Leica fluorescence MDR microscope (40x magnification). Pictures were recorded with a DC500 digital camera microscope using Leica LAS X software with the following exposure times: Alexa 488/FITC: 0.7 s; mCherry: 0.7 s; Hoechst: 0.136 s (1x gain).

### Immunizations and challenge with *P. berghei* parasites

*Pb*-Luci and genetically attenuated spz (*Pb*Δ*b9*Δ*slarp* and *Pb*Δ*mei2*Δ*lisp2*) were obtained through hand-dissection of salivary glands from infected *A. stephensi* mosquitoes. Radiation-attenuated sporozoites (RAS) were obtained through exposure of freshly dissected *Pb*-Luci spz to *γ*-radiation (16,000 rad in a Gammacell 3000 ELAN irradiator). Chemoprophylaxis and spz (CPS) immunization was performed by the administration of *Pb*-Luci spz and chloroquine, which was used as a schizonticidal drug to eliminate blood stage parasites. Chloroquine (Chloroquine diphosphate salt, Sigma-Aldrich) was prepared at a concentration of 35 mg/kg mouse body weight in PBS and administered daily through intraperitoneal injection for four days after each immunization. C57BL/6J mice were anaesthetized using isoflurane (Steve) and the immunizing parasites were inoculated at defined dosages through retro-orbital I.V. injection, as per the schedules described. Naïve and immunized mice were challenged through I.V. injection of 30 K *Pb*-Luci spz.

### Assessment of parasitemia

The presence of luciferase-expressing erythrocytic stage parasites was monitored daily, from day 2 after the infectious challenge, employing the Firefly Luciferase Assay Kit 2.0 (Biotium, Fremont, CA, USA), as described in ref. ^33^. Briefly, 5 μl of blood was collected from the mouse’s tail vein into 45 μl of lysis buffer and preserved at −20 °C. Parasite bioluminescence was measured by adding 50 μl of D-Luciferin dissolved in luciferase assay buffer to 15 μl of the blood lysate, using blood from non-infected mice as an internal control. Luminescence was immediately measured on a microplate reader (Tecan M200, Switzerland).

### Assessment of experimental cerebral malaria (ECM) symptoms

ECM symptoms were monitored daily using the rapid murine and behavior scale (RMCBS) score, as described in ref. ^69^. Mice with RMCBS score equal or below 5/20 were classified as displaying ECM symptoms and euthanized immediately through overdose of the isoflurane (Steve) inhalant anesthetic.

### Quantification of *P. berghei* liver load

Mouse parasite liver load was assessed at specified time-points post inoculation of the different surrogates of WSpz formulations or at 48 h after *Pb*-Luci infectious challenge and quantified by RT-qPCR, as previously described^70^. Liver lobes collected for RT-qPCR analysis were homogenized in 3 mL of denaturing solution (4 M guanidine thiocyanate; 25 mM sodium citrate pH 7; 0.5% w/v *N*-lauroylsarcosine and 0.7% v/v β-mercaptoethanol in DEPC-treated water). Total RNA was extracted from liver homogenates using the TripleXtractor Direct RNA kit (Grisp), according to the manufacturer’s instructions. The concentration of RNA in each sample was assessed by measurement of absorbance at 260 nm on a NanoDrop 2000 spectrophotometer. Complementary DNA (cDNA) was synthesized from 1 μg of RNA using the NZY First-Strand cDNA synthesis kit (NZYTech), according to the manufacturer’s instructions. The cDNA was synthesized in a Biometra Personal thermocycler employing the following parameters: 25 °C for 10 min, 55 °C for 30 min and 85 °C for 5 min. The RT-qPCR reaction was performed in a total volume of 10 μl in a Viia 7 Real-Time PCR system (Applied Biosystems) using the iTaq™ Universal SYBR® Green kit (BioRad). Parasite load was quantified using *Pb* 18S rRNA-specific primers. Mouse housekeeping gene hypoxanthine-guanine phosphoribosyltransferase (*Hprt*) expression was used for normalization. Primers used in the RT-qPCR are listed in Supplementary Table 1. Analysis of RT-qPCR data was performed using the delta-delta C_T_ relative quantification method^71^.

### Liver histopathology

Livers collected from non-immunized (challenge controls) and immunized mice 48 h after the infectious challenge with *Pb*-Luci, as well as from naïve controls were formalin-fixed in neutral buffered formalin, paraffin-embedded, cut in 3 µm sections, and stained with Harris Hematoxylin (Bio-Optica, Milan, Italy) and Eosin Y (Sigma, Missouri, USA). Tissue sections were analyzed by a pathologist using a Leica DM2000 microscope coupled to a Leica MC170 HD microscope camera (Leica Microsystems, Wetzlar, Germany). This work was supported by the Comparative Pathology Unit of the Instituto de Medicina Molecular and by the Histology service of Instituto Gulbenkian da Ciência.

### Biochemical assessment of liver pathology

Blood was drawn by cardiac puncture and, after clotting, samples were centrifuged for 10 min at 2000 × *g*. Serum was transferred to a fresh tube and the following parameters were determined (DNAtech, Lisbon, Portugal): total bilirubin, glucose, alanine aminotransferase (ALT) and aspartate transaminase (AST).

### Isolation of spleen and liver leukocytes

For tissue analysis, mice were sacrificed 48 h after the infectious challenge with *Pb*-Luci and extensively perfused with PBS 1x through the heart’s left ventricle. The spleen and liver were collected and homogenized using 70 and 100 μm cell strainers, respectively, in PBS containing 2% FBS (FACS buffer). The homogenized suspensions were centrifuged at 400 × *g* for 5 min at RT and the resulting cell pellet was depleted of RBCs by incubation in ammonium-chloride-potassium (ACK) solution for 3 min at RT and subsequent inactivation with FACS buffer. Separation of liver leukocytes involves an additional step using 35% (v/v) of Percoll gradient medium (Sigma) diluted in non-supplemented RPMI (Gibco-Thermo Fisher Scientific, Waltham, MA USA), followed by centrifugation at 1360 × *g* for 20 min without brake at RT. Finally, spleen and liver leukocyte suspensions were centrifuged at 400 × *g* for 5 min at 6 °C and resuspended in FACS buffer for subsequent staining.

### Flow cytometry analysis of spleen and liver leukocytes

One million leukocytes from each mouse were plated in 96-well plates, centrifuged at 845 × *g* for 5 min at 4 °C and Fc-blocked by incubation with anti-CD16/CD32 (clone 93; eBioscience/Thermo Fisher Scientific, Waltham, MA, USA; catalog #14-0161-92; 1:50 dilution) for 10 min at 4 °C. Spleen and liver leukocytes were then surface-stained for 20 min at 4 °C with a mix of antibodies and a Fixable Viability Dye (eBioscience, Thermo Fisher Scientific, Waltham, MA, USA) for exclusion of dead cells. The following conjugated anti-mouse monoclonal antibodies (and respective clones) were employed: CD62L FITC (MEL-14; catalog #104405; 1:200 dilution), CD3 PerCPCy5.5 (145-2C11; catalog #100217; 1:100 dilution), TCR*γδ* BV421 (GL3; catalog #118119; 1:100 dilution), CD8 Brilliant Violet (BV) 510 (GK1.5; catalog #100751; 1:200 dilution), CD4 BV605 (RM4-5; catalog #100547; 1:200 dilution), CD69 BV650 (H1.2F3; catalog #104541; 1:100 dilution), NK1.1 BV711 (PK136; catalog #108745; 1:100 dilution), CD44 BV785 (IM7; catalog #103041; 1:300 dilution), CXCR3 APC (CXCR3-173; catalog #126511; 1:100 dilution), CD45 Alexa Fluor (AF) 700 (30-F11; catalog #103127; 1:300 dilution), KLRG1 PE (MAFA; catalog #138407; 1:400 dilution) and CD127 PE-Dazzle 594 (A7R34; catalog #135031; 1:100 dilution), all from BioLegend (San Diego, CA, USA). Cells were acquired in a BD LSR Fortessa X-20 cytometer and analyses were performed within live, single (based on FSC-a vs. FSC-H parameters) CD45+ leukocytes using FlowJo v10 (FlowJo, BD). For the clustering and visualization of high-dimensional data, equivalent numbers of live, single CD45+ cells from each condition were concatenated. Clustering was performed using X-shift (number of clusters determined by the algorithm) and data are presented using the dimensionality reduction method TriMAP (large-scale dimensionality reduction using triplets).

### Statistical analyses

Statistical analyses were performed using the GraphPad Prism 9 (GraphPad Software, Inc) and results are presented as mean ± SD. All datasets were analyzed for normality with the Shapiro–Wilk or the Kolmogorov–Smirnov normality tests prior to statistical analyses. Data on spz numbers and in vitro EEF size were analyzed employing the unpaired Student’s *t* test. All the other data were compared using the Kruskal–Wallis with the Dunn’s or Tukey’s multiple comparison post-test. Survival curve analysis was carried out by Kaplan–Meier plots and *P* values were calculated using the Log-rank (Mantel–cox) test. Significance of the differences observed is indicated in each figure, ns – not significant, *^,#^*P* < 0.05; **^,##^*P* < 0.01; ***^,###^*P* < 0.001 and ****^,####^*P* < 0.0001.

### Reporting summary

Further information on research design is available in the Nature Research Reporting Summary linked to this article.

### Supplementary information

Supplementary Material

REPORTING SUMMARY

## Data Availability

All data needed to evaluate the conclusions in this paper are present in the paper or the Supplementary Materials.
