# Supplementary material for: The effect of dosage on the protective efficacy of whole-sporozoite formulations for immunization against malaria
Source: NPJ Vaccines. 2023 Nov 24;8:182. doi: 10.1038/s41541-023-00778-9 (PMC10667361; doi:10.1038/s41541-023-00778-9)
Supplement: Supplementary file 1 — Supplementary Material [file 41541_2023_778_MOESM1_ESM.pdf]

Primer ID	Gene ID	Sequence	Product (bp)	Description
		TTTGCTGACCTGCTGGATTAC	N/A	Forward m*hprt*
		CAAGACATTCTTTCCAGTTAAAGTTG	N/A	Reverse m*hprt*
		AAGCATTAAATAAAGCGAATACATCCTTAC	N/A	Forward *pb18S*
		GGAGATTGGTTTTGACGTTTATGTG	N/A	Reverse *pb18S*
p13		AATGAAGCGACGTATCGACC	1081	Forward *tgdhfr*
p14		TGATGCGTTCCTTGTTGAGG		Reverse *tgdhfr*
p15		CATATAAACACAAATGATGTTTTTTC	1543	Forward 3' *lisp2* integration
p16		GAAGAAGTATGACCATACGC		Reverse 3' *lisp2* integration
	PBANKA_1003000	CAGATTCGGCTTATCCATCTC	966	Forward *lisp2* ORF
	PBANKA_1003000	CACCATTGATTTGTTCCTCAC		Reverse *lisp2* ORF

Bp – base pairs; m – mouse; *pb – Plasmodium berghei*; *tg – Toxoplasma gondii*; N/A – non applicable

Supplementary Table 1. List of primers used in this study.

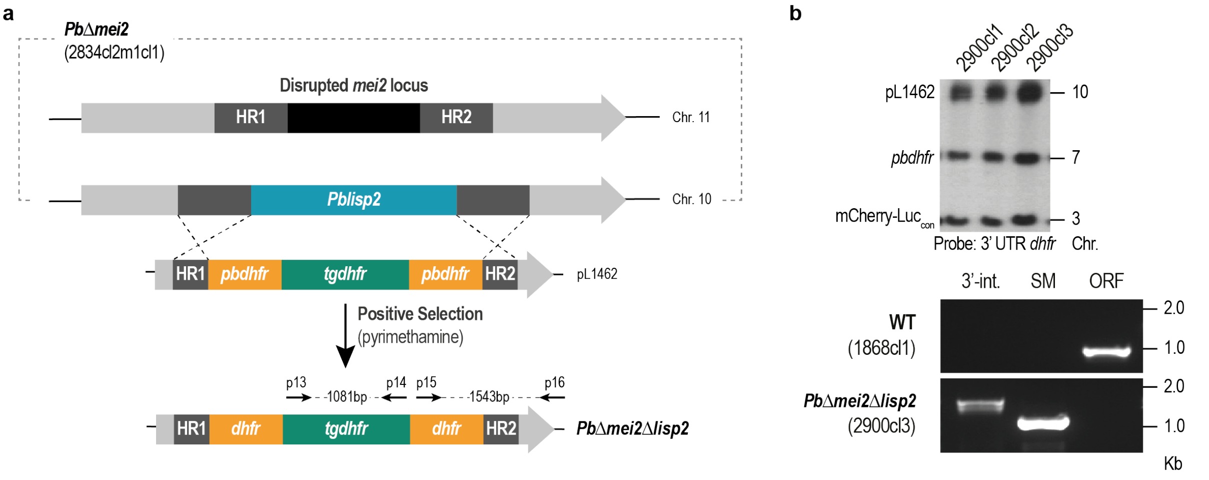

c

Parasite line	Sporozoite number ($x10^3$)[1]
WT	20.8 ± 4.2
*Pb*Δ*mei2*Δ*lisp2*	14.9 ± 7.7

[1]Mean number of sporozoites per mosquito

d

Parasite line	Sporozoite dose ($x10^4$)	No. of mice patent	No. of days to patency
WT	5	3/3	5
*Pb*Δ*mei2*Δ*lisp2*	5	0/6	n.a.
	20	0/10	n.a.
	30	0/3	n.a.

n.a. - not applicable

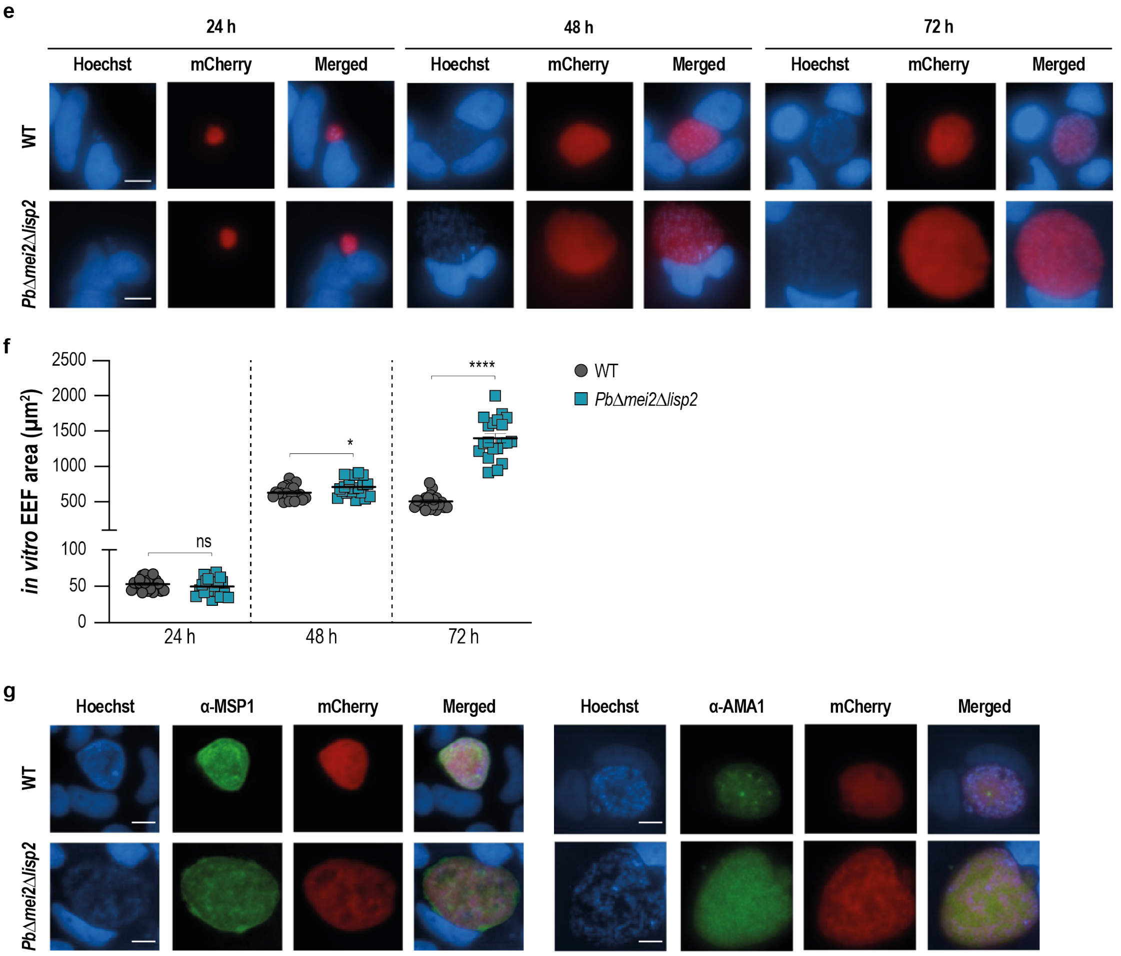

Supplementary Figure 1: Generation, genotyping, and phenotyping of the double gene-deletion mutant *Pb*Δ*mei2*Δ*lisp2*. **(a)** Schematic representation of the introduction of the *tgdhfr*

SM cassette into the *Pblisp2* locus of the single gene-deletion mutant *PbΔmei2* (line 2834cl2m1cl1). To delete the *lisp2*, the gene-deletion construct pL1462, which contains the *tgdhfr* flanked by the *pbdhfr* promoter region and the 3'-UTR of *pbdhfr*, was used. This construct integrates into the *lisp2* locus by double cross-over homologous recombination at the *lisp2* homology regions (HR1, HR2). Positive selection with pyrimethamine selects for parasites that have the *lisp2* coding sequence replaced by the SM cassette of the pL1462 construct, resulting in the *PbΔmei2Δlisp* parasite line. Location of primers (p) used for PCR analyses and sizes of PCR products are shown. Details of primers are shown in **Supplementary Table 1**. Chr. – Chromosomes. **(b)** Southern analysis of PFG-separated chromosomes (top) and diagnostic PCR (bottom) confirm correct integration of construct pL1462 in *PbΔmei2* (2834cl2m1cl1) parasite line. PCR shows the presence of the *tgdhfr* SM (primers p13/p14) and 3' integration of *lisp2* PCR (3'-int; primers p15/p16). **(c)** Salivary gland spz numbers in WT- and *PbΔmei2Δlisp2*-infected mosquitoes. **(d)** Blood stage infections in C57BL/6J mice infected through retro-orbital I.V. injection of a single dose of either WT or *PbΔmei2Δlisp2* spz. **(e)** Representative live images of WT and *PbΔmei2Δlisp2* EEFs developing in Huh7 cells at different time-points post infection. EEFs express cytoplasmic mCherry (red) under the control of the constitutive *hsp70* promoter. Nuclei of both the parasites and Huh7 cells were stained with Hoechst (blue). Scale bar: 10 µm. **(f)** EEF area at 24, 48 and 72 hpi of Huh 7 cells with spz of either WT (grey dots) or *PbΔmei2Δlisp2* (blue squares), assessed by live imaging of mCherry-expressing parasites. Data are expressed as mean ± SD and were compared using the unpaired Student's *t*-test (ns, not significant; *P < 0.05; ****P < 0.0001). **(g)** Representative immunofluorescence microscopy images of WT and *PbΔmei2Δlisp2* parasites expressing merozoite proteins MSP1 and AMA1 at 72 hpi of Huh7 cells. Immunofluorescence staining with anti-MSP1 (green) and anti-AMA1 (green), confirms the expression of both proteins by the LA-GAP. Liver stage parasites express cytoplasmic mCherry (red). Nuclei of both the parasites and Huh7 cells were stained with Hoechst (blue). Scale bar: 10 µm.

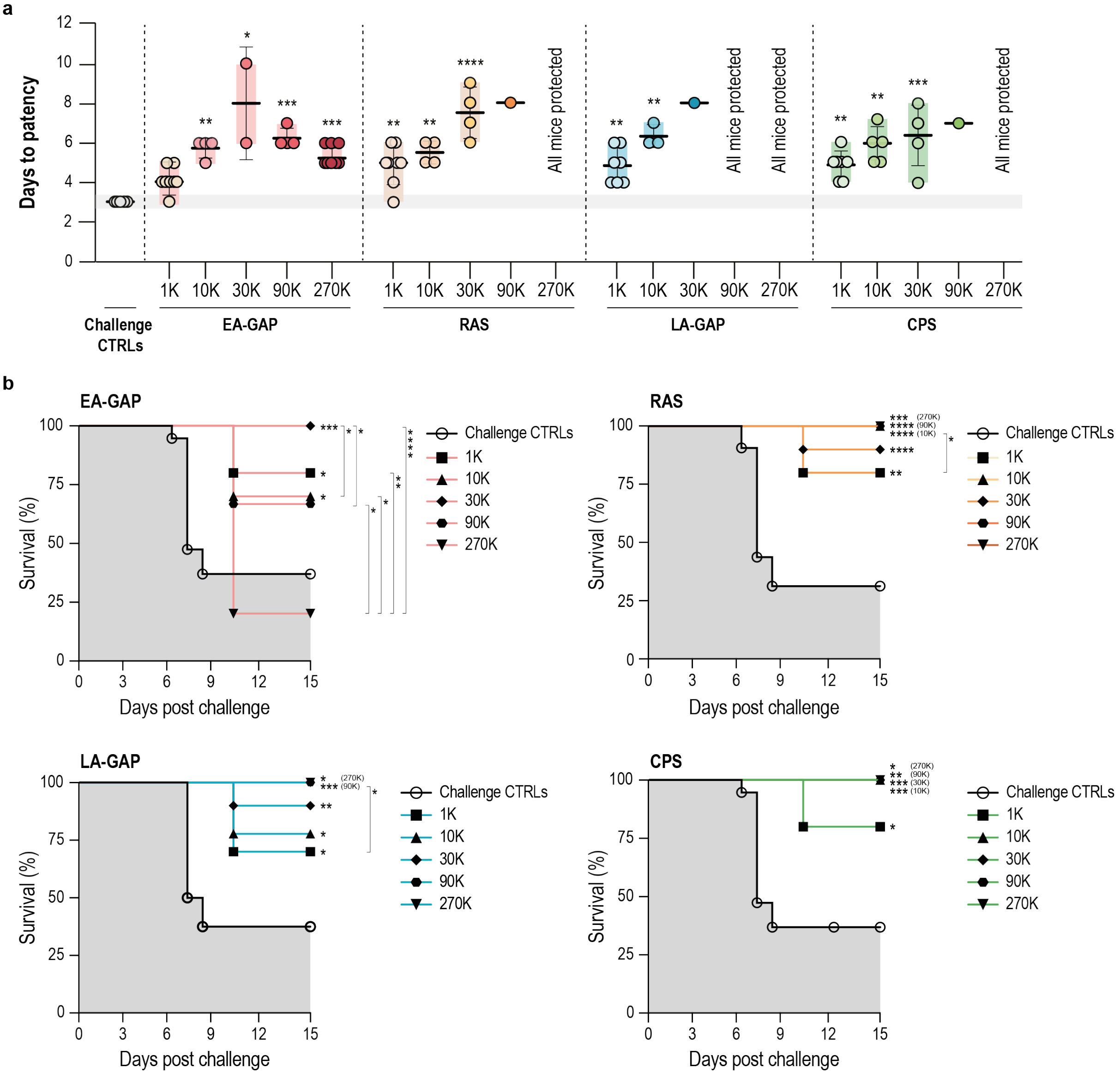

Supplementary Figure 2: Prepatent period and protection from experimental cerebral malaria (ECM) of C57BL/6J mice following a P2B immunization with different dosages of the various WSpz formulations. **(a)** Days to patency for challenge (non-immunized) controls (grey circles) and immunized but non-protected mice (coloured circles) are presented as the number of days until the detection of blood stage parasites, as measured through a bioluminescence assay. Data are expressed as mean ± SD and were compared using the Kruskal-Wallis test with Dunn's multiple comparison post-test whenever the number of non-protected mice allowed for sufficient statistical power (*P < 0.05; **P < 0.01; ***P < 0.001; ****P < 0.0001). **(b)** Kaplan-Meier survival plots showing the percentage of mice which developed ECM for each WSpz dosage and immunization approach (n = 5-20 mice per immunization dosage from 1-4

independent experiments). Statistically significant differences relative challenge controls (* next to symbols) or between experimental groups (* next to the lines) were calculated by Log-rank (Mantel-Cox) test. Signs of ECM were monitored daily between days 5 and 10 post challenge.

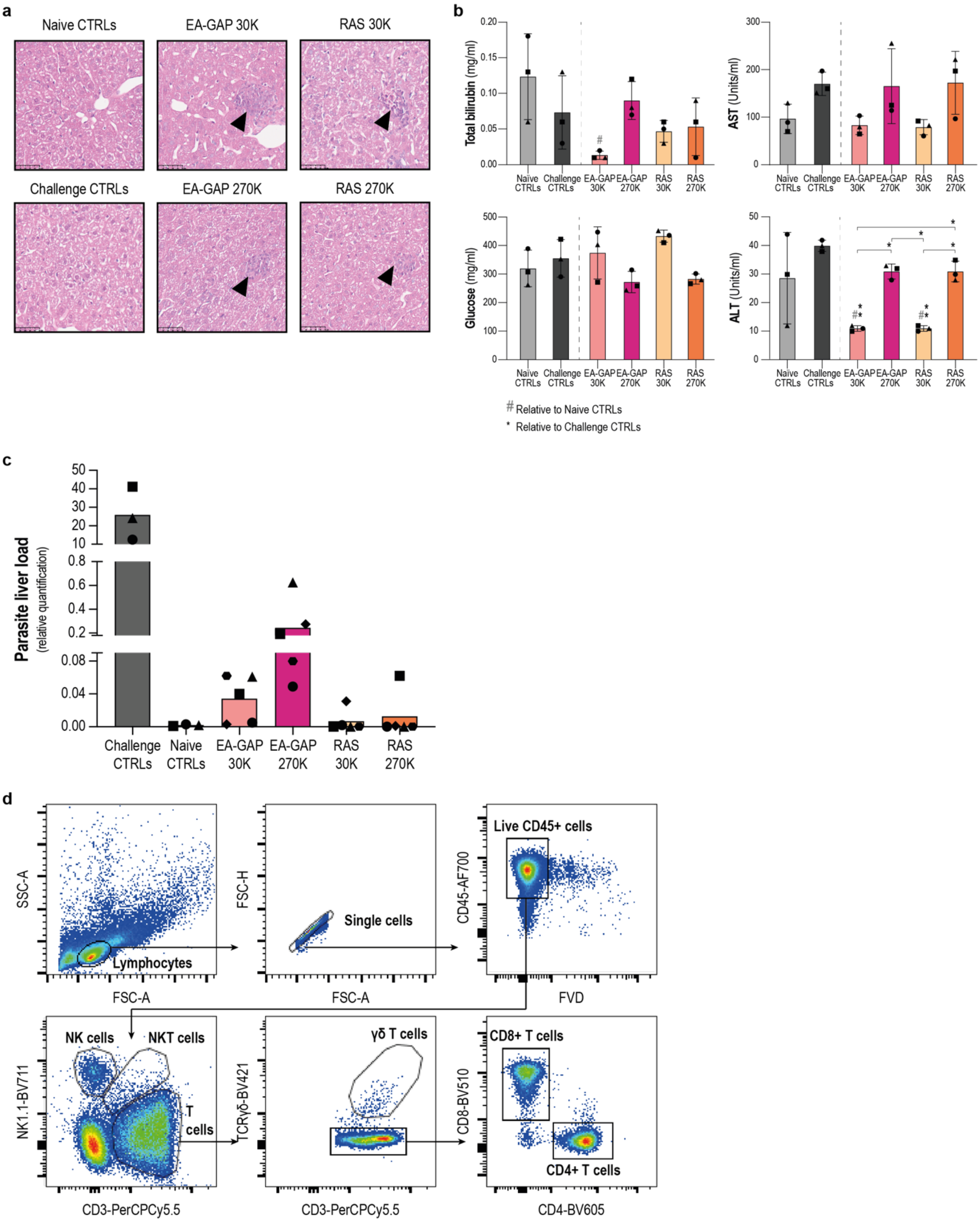

Supplementary Figure 3: Liver pathology and immune population analyses of the spleens and livers of C57BL/6J mice immunized with either 30 or 270K EA-GAP or RAS WSpz following a P2B immunization regimen. **(a)** Representative microphotographs of haematoxylin- and eosin-stained liver sections collected from non-immunized (challenge controls) and immunized mice 48 h after *Pb*-Luci infectious challenge as well as from naïve controls (n = 3-

5 mice per group). Arrows indicate inflammatory cell infiltrates. Scale bar: 50 µm. **(b)** Quantification of the levels of metabolites (total bilirubin, glucose, aspartate aminotransferase, AST, alanine aminotransferase, ALT) in the serum collected from non-immunized (challenge controls) and immunized mice 48 h after *Pb*-Luci infectious challenge as well as from naïve controls (n = 3 mice per group). Data are expressed as mean $\pm$ SD. Statistically significant differences relative to naïve control mice (#), relative to challenge controls (* above the bars) or between experimental groups (* above the lines) were assessed through the Kruskal-Wallis test with Dunn's multiple comparison post-test ($^{*,\#}P < 0.05$; $^{**,\#\#}P < 0.01$). **(c)** Parasite liver load 48 h post challenge of C57BL/6J mice immunized either with 30 or 270K EA-GAP or RAS WSpz and compared to the corresponding challenge control mice. Symbols represent the individual values of each mouse and bars indicate the mean. **(d)** Representative gating strategy applied for the flow cytometric analysis of immune populations of the spleen and liver. FVD: Fixable viability dye.

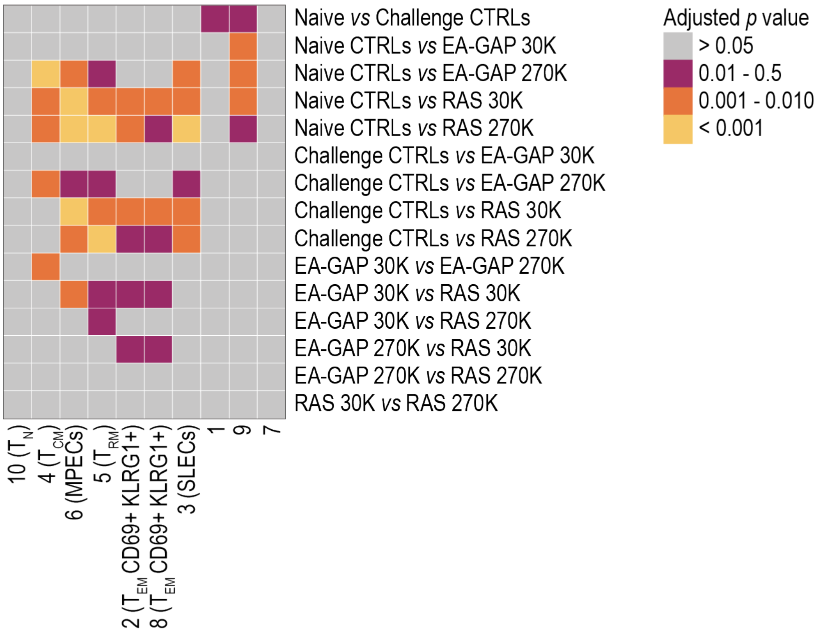

Supplementary Figure 4: Clustering analysis of the CD8+ T cell pool in the livers of C57BL/6J mice immunized with either 30 or 270K EA-GAP or RAS WSpz following a P2B immunization regimen. Heatmap of the statistical analysis performed for Fig. 4D on the number of cells within each cluster and in each experimental condition.
