# Supplementary material for: The effect of dosage on the protective efficacy of whole-sporozoite formulations for immunization against malaria
Source: NPJ Vaccines. 2023 Nov 24;8:182. doi: 10.1038/s41541-023-00778-9 (PMC10667361; doi:10.1038/s41541-023-00778-9)
Supplement: Supplementary file 2 — REPORTING SUMMARY [file 41541_2023_778_MOESM2_ESM.pdf]

nature portfolio

Corresponding author(s): Miguel Prudêncio; António M. Mendes

Last updated by author(s): Nov 2, 2023

Reporting Summary

Nature Portfolio wishes to improve the reproducibility of the work that we publish. This form provides structure for consistency and transparency in reporting. For further information on Nature Portfolio policies, see our Editorial Policies and the Editorial Policy Checklist.

Statistics

For all statistical analyses, confirm that the following items are present in the figure legend, table legend, main text, or Methods section.

n/a	Confirmed	
☐	☒	The exact sample size (*n*) for each experimental group/condition, given as a discrete number and unit of measurement
☐	☒	A statement on whether measurements were taken from distinct samples or whether the same sample was measured repeatedly
☐	☒	The statistical test(s) used AND whether they are one- or two-sided<br>*Only common tests should be described solely by name; describe more complex techniques in the Methods section.*
☒	☐	A description of all covariates tested
☐	☒	A description of any assumptions or corrections, such as tests of normality and adjustment for multiple comparisons
☐	☒	A full description of the statistical parameters including central tendency (e.g. means) or other basic estimates (e.g. regression coefficient) AND variation (e.g. standard deviation) or associated estimates of uncertainty (e.g. confidence intervals)
☒	☐	For null hypothesis testing, the test statistic (e.g. *F*, *t*, *r*) with confidence intervals, effect sizes, degrees of freedom and *P* value noted<br>*Give P values as exact values whenever suitable.*
☒	☐	For Bayesian analysis, information on the choice of priors and Markov chain Monte Carlo settings
☒	☐	For hierarchical and complex designs, identification of the appropriate level for tests and full reporting of outcomes
☒	☐	Estimates of effect sizes (e.g. Cohen's *d*, Pearson's *r*), indicating how they were calculated

Our web collection on statistics for biologists contains articles on many of the points above.

Software and code

Policy information about availability of computer code

Data collection	LSRFortessa X-20 (BD Biosciences); QuantStudio 5 Real-Time PCR systems (Applied Biosystems); Leica fluorescence MDR microscope (Leica); Microplate Reader TECAN Infinite M200 (Tecan); Leica MC170 HD microscope camera (Leica Microsystems).
Data analysis	FlowJo v10 software (FlowJo, BD); QuantStudio Design and Analysis Software v1.4.2 (Applied Biosystems); ImageJ 1.53 (NIH); GraphPad Prism software (version 6.01).

For manuscripts utilizing custom algorithms or software that are central to the research but not yet described in published literature, software must be made available to editors and reviewers. We strongly encourage code deposition in a community repository (e.g. GitHub). See the Nature Portfolio guidelines for submitting code & software for further information.

Data

Policy information about availability of data

All manuscripts must include a data availability statement. This statement should provide the following information, where applicable:

- Accession codes, unique identifiers, or web links for publicly available datasets
- A description of any restrictions on data availability
- For clinical datasets or third party data, please ensure that the statement adheres to our policy

All data needed to evaluate the conclusions in this paper are present in the paper or the Supplementary Materials.

Human research participants

Policy information about studies involving human research participants and Sex and Gender in Research.

Reporting on sex and gender	N/A
Population characteristics	N/A
Recruitment	N/A
Ethics oversight	N/A

Note that full information on the approval of the study protocol must also be provided in the manuscript.

Field-specific reporting

Please select the one below that is the best fit for your research. If you are not sure, read the appropriate sections before making your selection.

☒ Life sciences ☐ Behavioural & social sciences ☐ Ecological, evolutionary & environmental sciences

For a reference copy of the document with all sections, see nature.com/documents/nr-reporting-summary-flat.pdf

Life sciences study design

All studies must disclose on these points even when the disclosure is negative.

Sample size	Power analysis was performed to estimate the minimum number of experimental mice to maximize the chance of obtaining a statistically significant mean difference.
Data exclusions	No data were excluded form the analyses.
Replication	All attempts at replication were successful.
Randomization	Experimental animals were age- and sex-matched, and were randomly distributed by experimental groups.
Blinding	No blinding was performed, as blinding was not required for the type of analyses performed. All the experimental parameters measured and analyzed were deemed objective and not subject to any bias introduced by the researchers.

Reporting for specific materials, systems and methods

We require information from authors about some types of materials, experimental systems and methods used in many studies. Here, indicate whether each material, system or method listed is relevant to your study. If you are not sure if a list item applies to your research, read the appropriate section before selecting a response.

Materials & experimental systems

n/a	Involved in the study	
☐	☒	Antibodies
☐	☒	Eukaryotic cell lines
☒	☐	Palaeontology and archaeology
☐	☒	Animals and other organisms
☒	☐	Clinical data
☒	☐	Dual use research of concern

Methods

n/a	Involved in the study	
☒	☐	ChIP-seq
☐	☒	Flow cytometry
☒	☐	MRI-based neuroimaging

Antibodies

Antibodies used	The study included the following antibodies:<br>- Anti-CD16/CD32 (clone 93; eBioscience/Thermo Fisher Scientific)<br>- CD62L FITC (MEL-14; BioLegend)<br>- CD3 PerCPCy5.5 (145-2C11; BioLegend)<br>- TCRgd BV421 (GL3; BioLegend)<br>- CD8 BV510 (GK1.5; BioLegend)

- CD4 BV605 (RM4-5; BioLegend)
- CD69 BV650 (H1.2F3; BioLegend)
- NK1.1 BV711 (PK136; BioLegend)
- CD44 BV785 (IM7; BioLegend)
- CXCR3 APC (CXCR3-173; BioLegend)
- CD45 AF700 (30-F11; BioLegend)
- KLRG1 PE (MAFA; BioLegend)
- CD127 PE-Dazzle 594; BioLegend)
- Rabbit anti-Plasmodium yoelii MSP1
- Rat anti-Plasmodium falciparum AMA1
- Anti-rabbit IgG Alexa Fluor 488 (Invitrogen)
- Anti-rat IgG FITC (Thermofisher)

Validation

All the antibodies were optimized and validated (i.e. assay and species) by the supplier, more information on any validation statements can be found on the manufacturer's website.
- Anti-CD16/CD32 (clone 93; eBioscience/Thermo Fisher Scientific): https://www.thermofisher.com/antibody/product/CD16-CD32-Antibody-clone-93-Monoclonal/14-0161-82
- CD62L FITC (MEL-14; BioLegend): https://www.biolegend.com/nl-nl/products/fitc-anti-mouse-cd62l-antibody-384
- CD3 PerCPCy5.5 (145-2C11; BioLegend): https://www.biolegend.com/en-us/products/percp-cyanine5-5-anti-mouse-cd3-antibody-5596
- TCRgd BV421 (GL3; BioLegend): https://www.biolegend.com/nl-nl/products/brilliant-violet-421-anti-mouse-tcr-gamma-delta-antibody-7249
- CD8 BV510 (GK1.5; BioLegend): https://www.biolegend.com/nl-nl/products/brilliant-violet-510-anti-mouse-cd8a-antibody-7992
- CD4 BV605 (RM4-5; BioLegend): https://www.biolegend.com/nl-nl/products/brilliant-violet-605-anti-mouse-cd4-antibody-7627
- CD69 BV650 (H1.2F3; BioLegend): https://www.biolegend.com/nl-nl/products/brilliant-violet-650-anti-mouse-cd69-antibody-13310
- NK1.1 BV711 (PK136; BioLegend): https://www.biolegend.com/nl-nl/products/brilliant-violet-711-anti-mouse-nk-1-1-antibody-9576
- CD44 BV785 (IM7; BioLegend): https://www.biolegend.com/nl-nl/products/brilliant-violet-785-anti-mouse-human-cd44-antibody-7959
- CXCR3 APC (CXCR3-173; BioLegend): https://www.biolegend.com/nl-nl/products/apc-anti-mouse-cd183-cxcr3-antibody-4683
- CD45 AF700 (30-F11; BioLegend): https://www.biolegend.com/nl-nl/products/alexa-fluor-700-anti-mouse-cd45-antibody-3407
- KLRG1 PE (MAFA; BioLegend): https://www.biolegend.com/nl-nl/products/pe-anti-mouse-human-klrg1-mafa-antibody-6593
- CD127 PE-Dazzle 594; BioLegend): https://www.biolegend.com/nl-nl/products/pe-dazzle-594-anti-mouse-cd127-il-7ralpha-antibody-10321
- Anti-rabbit IgG Alexa Fluor 488 (Invitrogen): https://www.thermofisher.com/antibody/product/Goat-anti-Rabbit-IgG-H-L-Cross-Adsorbed-Secondary-Antibody-Polyclonal/A-11008
- Anti-rat IgG FITC (Thermofisher): https://www.thermofisher.com/antibody/product/Goat-anti-Rat-IgG-H-L-Secondary-Antibody-Polyclonal/31629

The following antibodies were validated in the articles referenced in the manuscript:
- Rabbit anti-Plasmodium yoelii MSP1 in Holder, A.A. and R.R. Freeman, Biosynthesis and processing of a Plasmodium falciparum schizont antigen recognized by immune serum and a monoclonal antibody. J Exp Med, 1982. 156(5): p. 1528-38.
- Rat anti-Plasmodium falciparum AMA1 in Kocken, C.H., et al., Precise timing of expression of a Plasmodium falciparum-derived transgene in Plasmodium berghei is a critical determinant of subsequent subcellular localization. J Biol Chem, 1998. 273(24): p. 15119-24.

Eukaryotic cell lines

Policy information about cell lines and Sex and Gender in Research

Cell line source(s)	HuH-7 is a well differentiated hepatocyte derived cellular carcinoma cell line deposited at the Japanese Collection of Research Bioresources under JCRB0403. The HepG2 cell line is a human hepatoma cell line deposited at ATCC under HB-8065.
Authentication	None of the cell lines were specifically authenticated for this study.
Mycoplasma contamination	All cell lines tested negative for mycoplasma contamination.
Commonly misidentified lines (See ICLAC register)	No commonly misidentified cell lines were used in the study.

Animals and other research organisms

Policy information about studies involving animals; ARRIVE guidelines recommended for reporting animal research, and Sex and Gender in Research

Laboratory animals	The study involved female OF1, male wild-type BALB/c and C57BL/6 mice (Mus musculus) aged 6-9 weeks produced by Charles River Laboratories (Leiden, Netherlands or Lyon, France).
Wild animals	The study did not involve wild animals.
Reporting on sex	Findings of this study only apply to one sex, as gender is not expected to play a significant role in the infection and immune analyses performed.

Field-collected samples	The study did not involve field-collected samples.
Ethics oversight	Instituto de Medicina Molecular - João Lobo Antunes' animal ethics committee Órgão Responsável pelo Bem-Estar Animal (ORBEA-iMM)

Note that full information on the approval of the study protocol must also be provided in the manuscript.

Flow Cytometry

Plots

Confirm that:

☒ The axis labels state the marker and fluorochrome used (e.g. CD4-FITC).

☒ The axis scales are clearly visible. Include numbers along axes only for bottom left plot of group (a 'group' is an analysis of identical markers).

☒ All plots are contour plots with outliers or pseudocolor plots.

☒ A numerical value for number of cells or percentage (with statistics) is provided.

Methodology

Sample preparation	The spleen and liver were collected and homogenised using 70 and 100 µm cell strainers, respectively, in PBS containing 2% FBS (FACS buffer). The homogenised suspensions were centrifuged at 400 x g for 5 min at RT and the resulting cell pellet was depleted of RBCs by incubation in ammonium-chloride-potassium (ACK) solution for 3 min at RT and subsequent inactivation with FACS buffer. Separation of liver leukocytes involves an additional step using 35% (v/v) of Percoll gradient medium (Sigma) diluted in non-supplemented RPMI (Gibco-Thermo Fisher Scientific, Waltham, MA USA), followed by centrifugation at 1,360 x g for 20 min without brake at RT. Finally, spleen and liver leukocyte suspensions were centrifuged at 400 x g for 5 min at 6 ? and resuspended in FACS buffer for subsequent staining.
Instrument	LSRFortessa X-20 (BD Biosciences)
Software	FlowJo v10 software (FlowJo, BD)
Cell population abundance	There was no sorting of cells.
Gating strategy	Analyses were performed within live, single (based on FSC-a vs. FSC-H parameters) CD45+ leukocytes. NK, NKT and T cells were identified based on NK1.1 and CD3 expression. Within the CD3+ NK1.1- T cell population, gd T and ab T cells were distinguished. TCRgd- and CD3+ T cells were further dissected into CD8+ and CD4+ T cells.

☒ Tick this box to confirm that a figure exemplifying the gating strategy is provided in the Supplementary Information.
